# Cellular senescence controls fibrosis in wound healing

**DOI:** 10.18632/aging.100201

**Published:** 2010-09-17

**Authors:** Joon-Il Jun, Lester F. Lau

**Affiliations:** Department of Biochemistry and Molecular Genetics, University of Illinois at Chicago, Chicago, IL 60607, USA

**Keywords:** CCN1, senescence, wound healing, integrin, inflammation

## Abstract

Mammalian wound healing involves the rapid synthesis and deposition of extracellular matrix (ECM) to maintain tissue integrity during repair. This process must be tightly controlled, as its deregulation may result in fibrosis, scarring, and loss of tissue function. Recent studies have uncovered an efficient and parsimonious mechanism for rendering fibrogenesis self-limiting in wound healing: in such diverse organs as the liver and skin, the myofibroblasts that initially proliferate and produce ECM are themselves eventually driven into senescence, blocking their further proliferation and converting them into matrix-degrading cells. Myofibroblast senescence in skin wounds is triggered by a dynamically expressed matricellular protein, CCN1/CYR61, which acts through integrin-mediated induction of oxidative stress. We propose that the onset of myofibroblast senescence is a programmed wound healing response that functions as a self-limiting mechanism for fibrogenesis, and this process may be regulated by the ECM microenvironment through the expression of CCN1/CYR61.

In a hostile environment rife with microbial invaders, mammals respond to wounding and tissue injury with a vigorous inflammatory response coupled to the rapid synthesis and deposition of extracellular matrix (ECM), thereby maintaining tissue integrity and providing defense against microbes while the wounded tissue is being repaired and remodeled. In virtually all mammalian organ systems, wound healing occurs similarly in three overlapping but distinct phases: inflammation, ECM deposition and tissue formation, and tissue remodeling [18,35,37]. Each of these steps must be tightly regulated for optimal wound healing. However, excessive ECM deposition may occur in wound repair, particularly in association with chronic injury and inflammation [15,38,43]. When excessive, non-functional ECM replaces parenchyma, the resulting fibrosis, scarring, and loss of tissue function may lead to deleterious consequences. For example, fibrotic scarring in the liver due to viral infections, in the lung from obstructive pulmonary disease, and in the heart following myocardial infractions can lead to organ failure and death. These types of dysfunctional wound healing adversely affect a large number of people worldwide, and inflict a significant burden on public health [18].

The principal cell type that contributes to the synthesis and deposition of ECM in healing wounds is the myofibroblast, which expresses α-smooth muscle actin and promotes wound contraction [43]. Myofibroblasts can be derived from a variety of sources, including differentiation of activated resident fibroblasts and recruited fibrocytes, and epithelial- and endothelial-mesenchymal transitions of epithelial and endothelial cells, respectively [14,43]. Whereas activated myo-fibroblasts proliferate and initially promote wound repair by producing ECM components, fibrosis may result when wound healing becomes chronic or if the ECM producing activity of myofibroblasts continues unchecked. However, the mechanism that keeps ECM production in balance with wound healing is poorly understood. Here we discuss the evidence indicating that myofibroblasts are driven into senescence at later stages of wound healing, thereby converting these ECM-producing cells into ECM-degrading cells, thus imposing a self-limiting control on fibrogenesis. In skin wound healing, myofibroblast senescence is triggered by the dynamically expressed matricellular protein CCN1 (also known as CYR61) through integrin signaling.

## Cellular senescence limits fibrosis during wound repair

First recognized in human fibroblasts experiencing replicative exhaustion in culture [19,20], cellular senescence is an essentially irreversible form of cell-cycle arrest that can be triggered by a variety of cellular damage or stress, including DNA damage, chromatin disruption, oncogene activation, oxidative stress, and telomere dysfunction [4,10]. Senescent cells remain viable and metabolically active, but are refractory to mitogenic stimulation. Another important feature of senescent cells is the expression of the senescence-associated secretory phenotype (SASP) or the senescence messaging secretome (SMS)[4,28,45], characterized by the increased expression of inflammatory cytokines/chemokines (e.g., IL1, IL6, IL8, MCP2, MCP4, MIP-1a, MIP-3a) and ECM degrading enzymes (e.g., matrix metalloproteinases [MMPs]), and downregulated expression of ECM components (e.g., collagen) [12,36]. Compelling evidence has established cellular senescence as an important mechanism of tumor suppression, which functions by blocking the proliferation of damaged cells that may be at risk of oncogenic transformation [3,9,13,29]. Paradoxically, the expression of SASP/SMS by senescent cells can also facilitate cancer progression by modifying the tissue microenvironment [11]. Therefore, senescent cells may have diverse and context-dependent effects on tissue pathologies. Although senescent cells have been found in various noncancerous pathologies and aging-related diseases, their roles in these contexts have not been thoroughly investigated [16,31].

Two recent studies have shown that senescent myofibroblasts accumulate as part of the normal process of tissue repair, and function to limit the extent of fibrogenesis associated with wound healing [22,26]. Upon damage in the liver, activated hepatic stellate cells are the primary source of myofibroblasts, which proliferate and produce matrix proteins to support hepatocyte proliferation and organ repair [2,32]. In chronic liver injuries, these cells are also responsible for excessive ECM production, leading to fibrosis and eventually cirrhosis. Krizhanovsky *et al.* showed that in mice subjected to repeated injections of carbon tetrachloride (CCl_4_), a protocol that induces liver damage and fibrosis, some of the ECM producing myofibroblasts eventually become senescent and express the SASP/SMS [26]. These senescent cells function to limit fibrosis in several ways: 1. they cease to proliferate, reducing the number of ECM producing cells; 2. they curtail the synthesis and promote the degradation of matrix components through the expression of SASP/SMS; and 3. they are eventually cleared by natural killer cells, thereby removing the myofibroblasts and accelerating the resolution of fibrogenesis and wound healing [26,44]. The expression of inflammatory cytokines as part of the SASP/SMS may also promote immune surveillance at the wound site [25,26]. Consistent with these interpretations, mice that are genetically defective for p53 and/or p16^INK4a^, which are critical for mediating senescence, suffer exacerbated fibrosis and delayed resolution of fibrosis in response to CCl_4_-induced injury.

**Figure 1.**
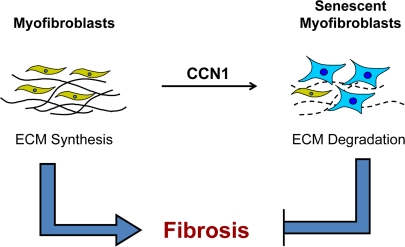
Myofibroblast senescence imposes self-limiting control on fibrogenesis during wound healing. Upon injury, myofibroblasts derived from activated fibroblasts and from other cell types proliferate and rapidly synthesize ECM to provide tissue integrity during repair. At later stages of wound healing, these ECM-producing myofibroblasts are themselves driven into senescence, whereupon they express an ECM-degrading phenotype characteristic of senescent cells. Therefore, fibrogenesis is self-limiting as myofibroblasts undergo senescence, thereby halting the proliferation of the ECM-producing cells and promoting ECM degradation. In cutaneous wound healing, senescence is triggered by the matricellular protein CCN1.

A similar mechanism of fibrosis control appears to operate in excisional cutaneous wound healing, which involves a tissue and mode of injury distinct from CCl_4_-induced liver damage [22]. During skin wound healing, recruited fibroblasts and differentiated myofibroblasts proliferate and deposit ECM to form the granulation tissue. Myofibroblasts are driven into senescence at later stages of wound healing, whereupon they cease to proliferate and upregulate the expression of matrix degrading enzymes (MMP2, MMP3, and MMP9) concomitant with downregulation of collagen and TGF-β, thereby exerting an anti-fibrotic effect [22]. Hence, the control of fibrogenesis during wound healing is efficient and parsimonious - the very cells that synthesize ECM in wound healing, the myofibroblasts, are themselves converted into matrix-degrading senescent cells to produce a self-limiting effect (Figure 1). These senescent cells may also promote tissue remodeling and clearance of the myofibroblasts during wound maturation. It is interesting to note that senescent cells are not required for wound healing *per se*, since healing occurs in mutant mice deficient in senescent cell accumulation [22,26].

## CCN1 controls cellular senescence in cutaneous wound healing

**Figure 2.**
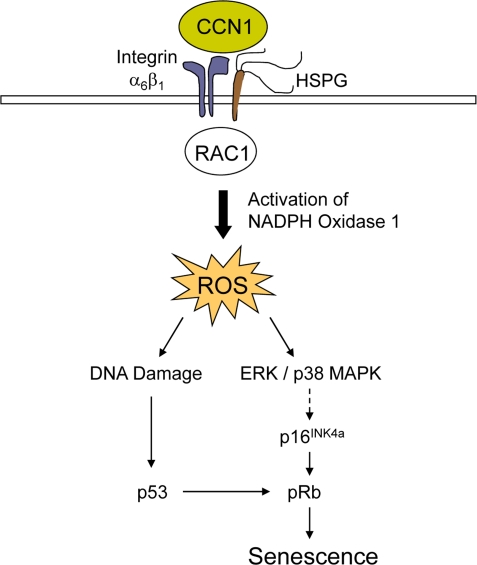
A mechanistic model for CCN1-induced senescence. The binding of CCN1 to its receptors in fibro-blasts, integrin α_6_β_1_and HSPGs, activates RAC1 and the RAC1-dependent NADPH oxidase 1 to generate a robust and sustained accumulation of ROS. This leads to a DNA damage response and activation of p53, as well as the ROS-dependent hyperactivation of ERK and p38 MAPK, leading to p16^INK4a^ induction [22]. Both p53 and p16^INK4a^ act upon pRb to induce senescence.

Whereas the factors that trigger senescence of activated stellate cells in CCl_4_-induced liver injury are currently unknown, senescence in cutaneous wounds is controlled by CCN1 (also known as CYR61), a matricellular protein dynamically expressed at sites of inflammation and wound healing [7]. Purified CCN1 protein can directly induce fibroblast senescence, both as a soluble factor and as an immobilized cell adhesion substrate [22]. Mechanistically, CCN1 induces fibroblast senescence through its direct binding to integrin α_6_β_1_ and cell surface heparan sulfate proteoglycans (HSPGs), thereby activating RAC1 and the RAC1-dependent NADPH oxidase 1 to trigger a robust and sustained accumulation of reactive oxygen species (ROS). Consequently, CCN1 induces DNA damage response and p53 activation, and triggers the ROS-dependent activation of p38 MAPK and ERK, which in turn activate the p16^INK4a^/pRb pathway to induce senescence (Figure 2). Both p53 and p16^INK4a^/pRb pathways contribute to CCN1-induced senescence [4,10]. Cell adhesion to CCN1 induces a much higher and more sustained level of ROS than cell adhesion to other ECM proteins such as collagen, fibronectin, and laminin, which do not induce senescence. The accumulation of a substantial level of ROS sustained for at least 10 hours appears necessary for efficient induction of senescence in fibroblasts [22]. A CCN1 mutant protein (DM) disrupted in its α_6_β_1_-HSPGs binding sites is unable to induce senescence or the SASP. Consistently, knockin mice in which the *dm* allele replaces the genomic *Ccn1* locus (*Ccn1^dm/dm^*) lack senescent cells in the granulation tissue and suffer exacerbated fibrosis during cutaneous wound healing [22]. Topical application of purified CCN1 protein to cutaneous wounds reverses these defects, further establishing the critical role of CCN1 in controlling myofibroblast senescence to limit fibrosis.

## Future questions and prospects

As the role of cellular senescence in wound healing and tissue repair is only beginning to be appreciated, many questions still remain. First, how broadly is cellular senescence invoked as a mechanism of fibrosis control? The observation that cellular senescence operates in both excisional skin wounds and toxin-induced liver injury, two different modes of wounding in disparate organ systems, suggests that senescence may be part of a general, programmed mechanism of fibrosis control in wound repair in diverse organs and tissues. Whether CCN1 functions to control senescence in contexts other than cutaneous wound healing is not yet known, although its high expression at many sites of inflammation and tissue injury suggests a role in disparate models of wound healing [7].

In addition to CCN1, other factors expressed in the wound microenvironment may also promote senescence. For example, overexpression of the plasminogen activator inhibitor-1 (PAI-1) is sufficient to drive fibroblasts into senescence *in vitro*[24]. PAI-1 knockout mice showed accelerated wound closure with diffused and unorganized collagen deposition, although whether PAI-1 controls senescence in healing wounds is currently unknown [6]. Interestingly, CCN1 can upregulate PAI-1, possibly through the activation of p53 [8]. Additionally, several secreted proteins such as insulin-like growth factor binding proteins (IGFBPs), cytokines such as IL6, and ligands of the chemokine receptor CXCR2 have been shown to mediate or reinforce senescence [1,23,27,34,41,42]. Some of these secreted factors are also involved in wound healing [17,21], although their potential role in myofibroblast senescence or fibrosis control remains to be explored.

Further investigation will be required to assess the role of cellular senescence in wound healing-related pathologies in humans. Senescent cells have been isolated from chronic and non-healing wounds such as pressure sores, diabetic ulcers, and venous ulcers, and may contribute to wound chronicity [39,40]. It is possible to postulate that excessive accumulation of senescent cells might have arisen from the enhanced expression of factors controlling senescence, such as CCN1 or PAI-1, as a measure to control fibrosis in chronic injury. Assessment of whether these senescence inducing factors are deregulated in chronic wounds may shed light on this issue. Senescent cells have also been found in various human pathologies associated with inflammation or injury repair, including atherosclerotic plaques [30], osteroarthritis [33], and benign prostatic hyperplasia [5]. Determining whether cellular senescence is invoked as a mechanism for fibrotic control in these contexts will be of interest. Further studies that identify the critical regulators of senescence in these pathologies, for which CCN1 is a candidate, may underscore potential signaling pathways for therapeutic intervention.
